# The anti-diabetic effects of betanin in streptozotocin-induced diabetic rats through modulating AMPK/SIRT1/NF-κB signaling pathway

**DOI:** 10.1186/s12986-021-00621-9

**Published:** 2021-10-16

**Authors:** Nasim Abedimanesh, Somayyeh Asghari, Kosar Mohammadnejad, Zahra Daneshvar, Soudeh Rahmani, Samaneh Shokoohi, Amir Hasan Farzaneh, Seyed Hojjat Hosseini, Iraj Jafari Anarkooli, Maryam Noubarani, Sina Andalib, Mohammad Reza Eskandari, Behrooz Motlagh

**Affiliations:** 1 Department of Nutrition, School of Medicine, Zanjan University of Medical Sciences, Zanjan, Iran; 2 Department of Clinical Nutrition, School of Nutritional Sciences and Dietetics, Tehran University of Medical Sciences, Tehran, Iran; 3 Department of Pharmacology and Toxicology, School of Pharmacy, Zanjan University of Medical Sciences, Zanjan, Iran; 4 Department of Physiology and Pharmacology, School of Medicine, Zanjan University of Medical Sciences, Zanjan, Iran; 5 Department of Anatomical Sciences, School of Medicine, Zanjan University of Medical Science, Zanjan, Iran; 6 Zanjan Pharmaceutical Nanotechnology Research Center (ZPNRC), Zanjan University of Medical Science, Zanjan, Iran; 7 Department of Clinical Biochemistry, School of Medicine, Zanjan University of Medical Sciences, Zanjan, Iran

**Keywords:** AMPK, SIRT1, NF-κB, Betanin, Diabetes, Rat

## Abstract

**Background:**

In the last few years, the effects of bioactive food components have received much attention because of their beneficial effects including decreasing inflammation, scavenging free radicals, and regulating cell signaling pathways. Betanin as a potent antioxidant has been previously reported to exhibit anti diabetic effects. The present study aimed to evaluate the effects of betanin on glycemic control, lipid profile, hepatic function tests, as well as the gene expression levels of 5′ adenosine monophosphate‑activated protein kinase (AMPK), sirtuin-1 (SIRT1), and nuclear factor kappa B (NF‑κB) in streptozocin (STZ) induced diabetic rats.

**Methods:**

Diabetes was induced in male Sprague–Dawley rats by intraperitoneal administration of STZ. Different doses of betanin (10, 20 and 40 mg/kg.b.w) was administered to diabetic rats for 28 days. Fasting blood glucose and serum insulin were measured. The histopathology of liver and pancreas tissue evaluated. Real-time PCR was performed to assess gene expression levels.

**Results:**

Treatment of diabetic rats with betanin (10 and 20 mg/kg.b.w) reduced FBG levels compared to the control diabetic rats (P < 0.001). Betanin at the dose of 20 mg/kg.b.w was most effective in increasing serum insulin levels (P < 0.001) improving glucose tolerance test (GTT) as well as improvement in lipid profile and liver enzymes levels. According to histopathologic assay, different damages induced by STZ to liver and pancreas tissues was largely eliminated by treatment with 10 and 20 mg/kg.b.w of betanin. Betanin also significantly upregulated the AMPK and SIRT1 and downregulated the NF-κB mRNA expression compared to the diabetic control rats (P < 0.05).

**Conclusion:**

Betanin could modulate AMPK/SIRT1/NF-κB signaling pathway and this may be one of its anti-diabetic molecular mechanisms.

## Background

Diabetes mellitus (DM) is one of the most critical health problems [1] which its complications reduce the patient’s quality of life [2]. The burden of DM has risen significantly [3] and it has been estimated that the number of diabetic people in the world will be rising to 642 million by 2040 [4]. The increasing morbidity and mortality rates observed among diabetic patients are majorly due to the high incidence and severity of diabetic complications due to lack of proper management and persistent hyperglycemia which results in derangements in metabolic processes such as glucose, lipid, and protein metabolism [5]. Although the precise etiopathogenesis of DM is not completely elucidated, oxidative stress, inflammatory factors, and autoimmune reactions have all been proposed as the major pathogenic risk factors for DM [6].

In the last few years, the effects of bioactive food components have received much attention because of their beneficial effects including decreasing inflammation, scavenging free radicals, and regulating cell signaling pathways [7]. Betalains, the natural pigments of red beetroot (*Beta vulgaris* L.) are presently gaining popularity for use as natural colorants in the food industry. Traditionally, red beetroots have been used to treat diabetes in many countries. Betanin is the main betalain that causes the red-violet color of beetroot and has been shown to exhibit anti-diabetes, anti-oxidative, and anti-inflammatory properties [8, 9].

Previous studies on rats with diabetes mellitus, oral betanin administrations positively regulated glycemic parameters and the activity of glycolytic enzymes such as glucokinase and pyruvate kinase. In addition, betanin administration significantly decreased gluconeogenic enzymes activity, which suggests that betanin could modulate carbohydrate metabolism [10, 11]. However, the exact effect of betanin in diabetes and the underlying cellular mechanisms have not been elucidated.

Adenosine 5′-monophosphate-activated protein kinase (AMPK) is a major energy sensor enzyme and a master regulator of metabolic homeostasis; hence, AMPK is considered as a key therapeutic target for the treatment of diabetes [12, 13]. AMPK can produce an anti-inflammatory effect and inhibit oxidative stress through the activation of the SIRT1-related pathway [14–16].

Sirtuins, a family of NAD^+^ dependent protein/histone deacetylases act as cellular metabolic sensors [17]. Of them, sirtuin-1 (SIRT1) has been shown to regulate various metabolic pathways including glucose homeostasis, lipid metabolism, insulin secretion and sensitivity, cellular aging, and apoptosis [18]. In addition, SIRT1 prevents diabetes by reducing oxidative stress and inflammation. It has been shown that overexpression of SIRT1 reduces insulin resistance [19] and its gene mutation may induce type 1 diabetes mellitus [20]. Therefore, alterations in SIRT1 gene expression may be associated with the pathogenesis of diabetes and its complications [19, 21].

AMPK and SIRT1 share many common target molecules and both regulate each other. The evidence suggests that the activation of AMPK signaling suppresses the expression of the nuclear factor-kappa B (NF-κB) by increasing the expression of SIRT1, thereby contributing to the protection against obesity, inflammation, and insulin resistance [15, 22]. SIRT1 and NF-κB have antagonist crosstalk in regulating inflammatory responses and metabolic disorders. SIRT1 shows an anti-inflammatory effect through inhibition of NF-κB activation. In contrast, NF-κB signaling, and inflammatory response can suppress the SIRT1 activity [23, 24]. Therefore, these pathways represent as potential targets for the development of therapies to prevent and reduce the incidence of metabolic disease complications and development.

The effects of several plant bioactive components on AMPK and SIRT1 activations have been demonstrated previously [25–27]. However, despite the available data on anti-diabetic properties of betanin, no evidence is available regarding possible effects of betanin on AMPK and/or SIRT1 expression levels in diabetes. Several previous studies reported the increased SIRT1 expression following betalains and betacyanins rich supplements [28, 29].

With this background, the aim of the current study was to investigate the effects of betanin on glycemic control, lipid profile, hepatic function tests, as well as histopathological findings of the liver and pancreas tissues and then to investigate whether betanin could modulate AMPK/SIRT1/NF-κB signaling pathway in STZ-induced diabetic rats.

## Materials and methods

### Chemicals and kits

STZ was purchased from Sigma Aldrich (Germany) and betanin was purchased from TCI (Japan). Rat insulin kit (ELISA) was provided from Bioassay Technology Laboratory (BT Lab), Shanghai Korain Biotech Co Ltd (China). The kits used for measuring aspartate aminotransferase (AST), alanine aminotransferase (ALT), triglyceride (TG), total cholesterol (TC), and high-density lipoprotein cholesterol (HDL-C) were obtained from Pars Azmoon, Iran. Ethanol, methanol, eosin, hematoxylin, and paraffin were purchased from MERCK (Germany). The total RNA isolation kit was provided from FAVORGEN (Taiwan). The cDNA synthesis kit was obtained from BioFACT (Korea) and SYBR Green Master Mix from TaKaRa (Japan).

Betanin was purchased from TCI America (CAS Number: 7659-95-2, Product number: B0397, Lot number: 25XUG).

### Experimental animals and design

A total of 42 male Sprague–Dawley rats (180–240 g) were used in this study. They were housed in a room with 12-h light/dark cycles, a temperature of 25 °C, and free access to the food and water. All procedures were according to the Ethics Committee of Zanjan University of Medical Sciences (IR.ZUMS.REC.1398.0134).

The experimental type 1 diabetes model was induced by the intraperitoneal injection of a single-dose of 60 mg/kg STZ freshly dissolved in sodium citrate buffer (pH: 4.5) [30–33]. After 72 h, blood samples were collected from the tail vein of the overnight fasted rats and glucose levels measured by a glucometer (GLUCOCARD, ARKRAY, Japan). Rats with FBG over 250 mg/dl were considered diabetic and included in the study.

The animals were divided randomly into seven groups (six rats in each group): group I: normal control + normal saline (0.5 mL/day, orally), group II: normal rats + betanin (40 mg/kg/day), group III: diabetic control + normal saline (0.5 mL/day, orally), group IV: diabetic rats + betanin (10 mg/kg/day, orally), group V: diabetic rats + betanin (20 mg/kg/day, orally), group VI: diabetic rats + betanin (40 mg/kg/day, orally), group VII: diabetic rats + glibenclamide as a positive control (5 mg/kg/day, orally).

The administration doses of betanin [11, 34] and glibenclamide [35, 36] were selected on the basis of previous studies. The fresh betanin solutions were orally administered daily using rat gavage for 28 days. Body weight were measured on the 0th and 28th days of the study. At the end of the study period, the animals fasted overnight and blood samples were collected from the portal vein after anesthetization with ether. The serum and whole blood samples were collected for further biochemical and gene expression examinations. Then the animals were sacrificed and their liver and pancreas were dissected out for histopathological examination.

### Measurement of fasting blood glucose and glucose tolerance test

On the 7th, 14th, 21th and 28th days of the study and after the overnight fasting, blood samples were obtained from the tail vein, and then FBG levels were measured by using a digital glucometer (GLUCOCARD, ARKRAY, Japan). For evaluating the glucose tolerance test (GTT), after the FBG measurement, a dose of 2 g/kg of glucose solution was administered orally to the rats on the 28th day of the study and then blood glucose levels were measured at the 60 and 120 min after the oral glucose load [37]. Glucose levels were measured by using a digital glucometer (GLUCOCARD, ARKRAY, Japan). The index of the area under the glycemic curve (AUC) was calculated in order to show the general increase in glucose concentrations after glucose consumption.

### Insulin measurement

On the 28th day of the study, the serum insulin levels were determined by rat insulin enzyme-linked immunosorbent assays kit (Bioassay Technology Laboratory, BT Lab, China) according to the instructions of manufacturer.

### Measurement of lipid profile and liver enzymes levels

Serum TG, TC, HDL-C, AST, and ALT levels were measured by a commercial kit (Pars Azmoon, Iran). LDL-C was calculated by the Friedewald [38] equation: LDL-Cholesterol (mg/dl) = [TC-HDL-(TG/5)].

### Histopathological examination

The pancreas and liver were dissected out and immediately rinsed with normal saline solution. These tissues were fixed in 10% formaldehyde for 48–72 h, embedded in paraffin, and were cut into 5 μm thick sections. Then, the sections were stained with hematoxylin and eosin (H&E) and examined by light microscopy.

### Real-time PCR

On the final day of the experiment, blood samples were collected from the rat’s retro-orbital plexus of the eye. First, the mRNA was extracted from whole blood samples and liver tissues by FavorPrep™ Blood/Cultured Cell Total RNA Mini Kit (FAVORGEN) according to the manufacturer’s protocol. The concentration of the extracted RNAs, as well as the 260/280 and 260/230 ratios, was determined to identify purity and contamination with chloroform and isopropanol using Nanodrop spectrophotometer (Thermo Scientific, USA). In addition, the integrity of RNAs was qualified by employing 2% agarose gel electrophoresis. The cDNA synthesis kit (2X RT-PCR Pre-Mix (Taq), BioFACT™) was applied to convert the extracted RNAs into cDNA. The gene expressions of AMPK, SIRT1, and NF-κB were measured using the Real-time PCR. For this purpose, 1 μl of synthesized cDNA was combined with 0.5 μl of each Forward/Reverse primer as well as deionized water and SYBR® Premix Ex TaqII PCR kit (TaKaRa). The PCR reaction was conducted using the ABI (StepOnePlus) system. The primers of target genes were designed using Gene Runner software and assessed with Oligo Analyser 3.1 software (Table 1). β-actin was additionally recruited as the housekeeping control gene. The relative expression level of target genes was normalized to β-actin and untreated control group levels. Ultimately, the fold change was calculated using the E^−ΔΔCt^ formula to represent the data. To ensure the quality of the products, final RT-PCR aliquots were run on 2% agarose gel electrophoresis and then visualized using a gel doc imaging system after being stained.

**Table 1:** Primer sequences

Gene	Sequence
*AMPK*	F. 5′AGATAGCTGACTTCGGACTCTCT3′ R. 5′AACCTCAGGACCCGCATACA3′
*NF-κB*	F. 5′AGAGCAACCGAAACAGAGAGG3′ R. 5′TTTGCAGGCCCCACATAGTT3′
*SIRT1*	F. 5′TGGACGAGCTGACCCTTGA3′ R. 5′TCCTGCGGATGTGGAGATT3′
*β-actin*	F. 5′TCACCCACACTGTGCCCATCTATGA3′ R. 5′CATCGGAACCGCTCATTGCCGATAG3′

### Statistical analysis

The statistical analysis of data was done by the SPSS software version 18. One-way analysis of variance (ANOVA) was done for evaluating significant differences between the mean values. The significant interrelation between the various groups was analyzed by Tukey’s multiple comparison test. *P*-value < 0.05 was considered statistically significant.

## Results

### Effect of betanin on body weight, fasting blood glucose, and serum insulin levels

All experimental groups showed a non-significant (P > 0.05) difference in their initial body weight. Diabetic control rats showed a significant (P < 0.01) decrease in body weight at the end of the study as compared to that of the normal control animals. Treatment of diabetic rats with 10 mg/kg and 20 mg/kg of betanin or glibenclamide exhibited an increase in body weight compared to diabetic control rats at the end of study but it was not significant (P > 0.05) (Table 2).

**Table 2:** Effect of betanin on body weight, FBG and serum insulin levels

Variables	Normal control	Normal + Betanin (40 mg/kg)	Diabetic control	Diabetic + betanin (10 mg/kg)	Diabetic + betanin (20 mg/kg)	Diabetic + betanin (40 mg/kg)	Diabetic + glibenclamide
*Body weight (g)*							
Initial	192.67 ± 32.07	196.80 ± 26.31	197.60 ± 45.00	197.00 ± 10.42	186.00 ± 23.53	182.20 ± 15.45	184.00 ± 8.06
Final	204.50 ± 29.90	205.20 ± 28.62	150.60 ± 16.47^††^	160.50 ± 9.25	176.60 ± 21.09	151.40 ± 19.97	182.80 ± 9.34
*FBG (mg/dl)*							
Day 7	86.00 ± 12.18	74.80 ± 7.36	383.60 ± 39.44^†††^	348.50 ± 11.03	355.00 ± 38.81	392.60 ± 52.32	334.00 ± 13.13
Day 14	94.83 ± 5.34	73.80 ± 8.58	413.60 ± 41.91^†††^	319.00 ± 5.89^ϕϕϕ^,**	317.80 ± 35.15^ϕϕϕ^,**	394.00 ± 43.52^###^	275.80 ± 12.85^ϕϕϕ^
Day 21	94.17 ± 9.28	75.80 ± 4.21	464.20 ± 52.01^†††^	283.25 ± 6.75^ϕϕϕ^,***	280.80 ± 27.52^ϕϕϕ^,***	404.60 ± 49.83^###^	228.00 ± 25.61^ϕϕϕ^
Day 28	92.67 ± 3.67	75.00 ± 2.91	499.80 ± 42.69^†††^	259.00 ± 3.37^ϕϕϕ^,***	235.00 ± 16.57^ϕϕϕ^,***	471.80 ± 20.56^###^	141.00 ± 21.29^ϕϕϕ^
Insulin (mIU/L)	24.67 ± 1.37	23.20 ± 2.77	9.00 ± 1.58^†††^	15.00 ± 1.82^ϕϕ^	19.00 ± 2.74^ϕϕϕ^^,^^#^,***	11.20 ± 1.92	14.40 ± 1.95^ϕϕ^

Data were expressed as mean ± SD

FBG: fasting blood glucose

^††^P < 0.01, ^†††^P < 0.001 versus normal control group

^ϕϕ^P < 0.01, ^ϕϕϕ^P < 0.001 versus diabetic control group

^#^P < 0.05, ^###^P < 0.001 versus diabetic + glibenclamide group

^**^P < 0.01, ***P < 0.001 versus diabetic + betanin 40 mg/kg group (differences among diabetic rats received betanin)

Hyperglycemia was recorded in diabetic control rats during the experimental period as compared to the normal control group. Treatment of diabetic rats with betanin (10 and 20 mg/kg) or glibenclamide significantly reduced FBG levels compared to the control diabetic rats at the end of the study (P < 0.001), while treatment with 40 mg/kg of betanin could not improve FBG levels significantly (P = 0.360). Glycemic lowering effect of betanin at the doses of 10 and 20 mg/kg was almost the same on days 14, 21, and 28 (Table 2).

Serum insulin level was measured at the end of study (day 28). There was a significant decrease in the insulin levels in diabetic control group compared to the normal control group (P < 0.001). Treatment with 20 mg/kg of betanin was shown to produce more significant (P < 0.001) effect on serum insulin level compared to the betanin at dose of 10 mg/kg (P = 0.003) and/or glibenclamide (P = 0.005), while treatment with 40 mg/kg of betanin in diabetic rats showed no significant effect on serum insulin level compared to the control diabetic rats (P = 0.634) (Table 2).

### Effect of betanin on GTT during 120 min

**Fig. 1:**
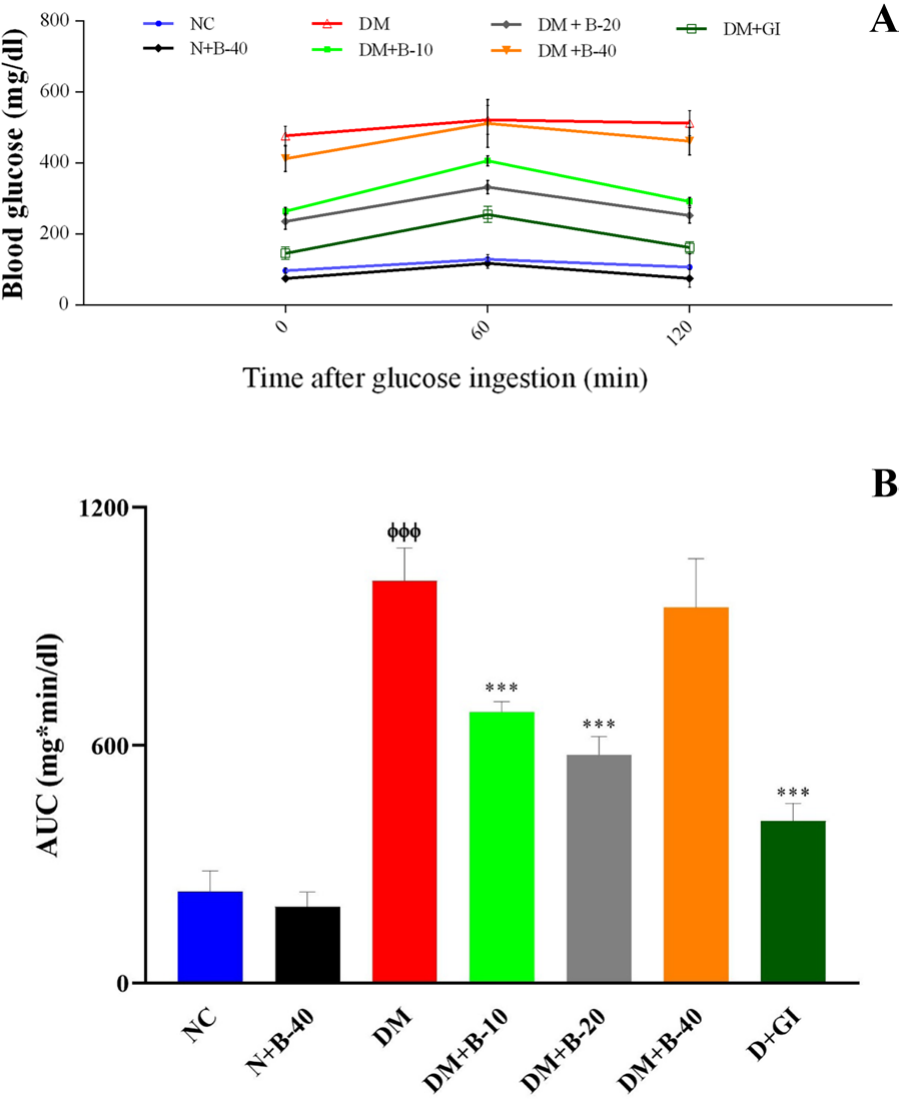
Effect of different doses of betanin (10, 20 and 40 mg/kg body weight) and Glibenclamide on oral glucose tolerance test (**A**) and area under the curve (AUC) after glucose load of rats (**B**). Data were presented by mean ± SD. NC: Normal Control, N + B-40: Normal rats + 40 mg/kg body weight betanin, DM: Diabetic Control, DM + B-10: Diabetic rat + 10 mg/kg body weight betanin, DM + B-20: Diabetic rat + 20 mg/kg body weight betanin, DM + B-40: Diabetic rat + 40 mg/kg body weight betanin, DM + GI: Diabetic rat + Glibenclamide. The number of rats in NC group was 6, in DM + B-10 was 4 and in all other study groups was 5.

The effect of betanin on GTT in control and experimental rats is shown in Fig. 1A. Blood glucose level was elevated to a peak value at 60 min and declined to near basal level at 120 min in betanin treated diabetic rats, whereas the blood glucose peak was noticed even after 60 min and remained high over the next 60 min in diabetic control rats. Similar pattern in blood glucose rise was observed in diabetic rats which received 40 mg/kg of betanin. After loading the glucose, it was observed that diabetic control rats showed the higher AUC_glucose_ values. Treatment with the different doses of the betanin (10 and 20 mg/kg) showed significantly lower AUC glucose values as compared to the diabetic control rats (P < 0.001) (Fig. 1B).

### Effect of betanin on lipid profile and liver enzymes levels

Lipid profile (TC, LDL-C, HDL-C, and TG) as well as liver enzymes levels (ALT and AST) were compared among control and experimental rats (Table 3). There were significant differences in TC, LDL-C, HDL-C (P < 0.001), and TG (P = 0.005) values between diabetic and normal control rats. Similar to glibenclamide, betanin significantly improved TC levels at the dose of 20 mg/kg compared to the diabetic control rats (P < 0.001). Significant decreases in LDL-C and TG as well as increase in HDL-C serum levels were seen in both diabetic rats treated with betanin at the doses of 10 and 20 mg/kg and the diabetic rats treated with glibenclamide in comparison to the diabetic control rats (P < 0.001). Betanin at the dose of 40 mg/kg had no significant effect on serum lipid profile variables (P > 0.05).

**Table 3:** Effect of betanin on serum lipid profile and liver enzymes levels

Variables	Normal control	Normal + Betanin (40 mg/kg)	Diabetic control	Diabetic + betanin (10 mg/kg)	Diabetic + betanin (20 mg/kg)	Diabetic + betanin (40 mg/kg)	Diabetic + glibenclamide
TC (mg/dL)	72.33 ± 5.35	70.60 ± 9.21	129.20 ± 8.11^†††^	115.50 ± 11.90^##^	98.40 ± 11.26^ϕϕϕ^,**	124.80 ± 7.59^###^	91.00 ± 11.25^ϕϕϕ^
LDL-C, (mg/dL)	15.83 ± 3.71	18.80 ± 5.40	57.00 ± 3.61^†††^	35.50 ± 2.64^ϕϕϕ^,***	30.00 ± 2.74^ϕϕϕ^,***	50.60 ± 5.94^###^	29.20 ± 4.82^ϕϕϕ^
HDL-C, (mg/dL)	43.83 ± 5.74	47.80 ± 4.76	23.40 ± 2.97^†††^	35.00 ± 3.56^ϕϕ^,***	35.20 ± 2.49^ϕϕ^,***	20.60 ± 2.07^###^	40.40 ± 2.41^ϕϕϕ^
TG, (mg/dL)	105.33 ± 52.83	91.20 ± 18.70	209.00 ± 63.28^††^	113.75 ± 17.56^ϕ^	98.60 ± 23.03^ϕϕ^	165.40 ± 59.47	99.60 ± 10.45^ϕϕ^
ALT, (U/L)	83.17 ± 12.32	82.40 ± 9.48	152.60 ± 10.97^†††^	138.75 ± 7.93	123.20 ± 9.68^ϕϕ^,**	148.80 ± 6.30	130.40 ± 12.70^ϕ^
AST, (U/L)	104.17 ± 17.52	105.60 ± 18.23	208.60 ± 25.28^†††^	141.25 ± 10.69^ϕϕϕ^,***	132.20 ± 10.64^ϕϕϕ^,***	198.00 ± 7.55^###^	140.20 ± 15.83^ϕϕϕ^

Data were expressed as mean ± SD

TC: total cholesterol, LDL-C: low-density lipoprotein cholesterol, HDL-C: high-density lipoprotein cholesterol, TG: triglyceride, ALT: alanine aminotransferase, AST: aspartate aminotransferase

^††^P < 0.01, ^†††^P < 0.001 versus normal control group

^ϕ^P < 0.05, ^ϕϕ^P < 0.01, ^ϕϕϕ^P < 0.001 versus diabetic control group

^###^P < 0.001 versus diabetic + glibenclamide group

^**^P < 0.01, ***P < 0.001 versus diabetic + betanin 40 mg/kg group (differences among diabetic rats received betanin)

Significant increases were identified in the levels of serum ALT and AST in the diabetic control group compared to the normal control group (P < 0.001). Treatment of diabetic rats with betanin (20 mg/kg body weight) or glibenclamide resulted in considerable reductions (P < 0.05) in the levels of ALT and AST when compared to the diabetic control group. Betanin at the dose of 10 mg/kg significantly reduced AST levels compared to the diabetic control rats (P < 0.001), while it had no significant effect on ALT levels.

### Histopathological findings

#### Liver

**Fig. 2:**
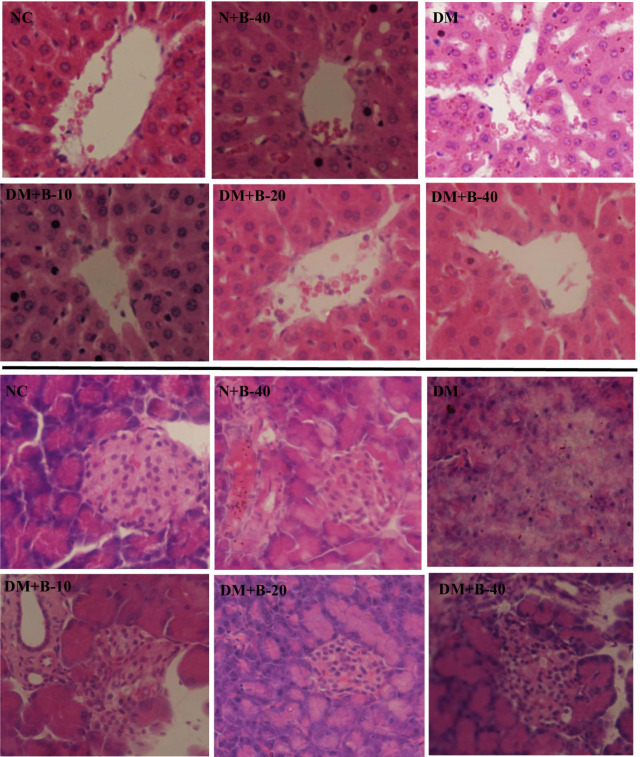
Histopathological findings of the liver and pancreas following treatments with different doses of betanin (10, 20 and 40 mg/kg.b.w) in SZT-induced diabetic and normal rats (the images above are related to liver tissue and the images below are related to pancreatic tissue)

Histopathological examination of the liver tissue showed no changes between the normal rats and those treated with high dose of betanin. The liver of the diabetic control rats showed disarrangement of hepatic strands, accumulation of fat droplets, inflammation, and dilation of sinusoids. While, treating diabetic rats with 10 and 20 mg/kg of betanin attenuated the hepatic inflammation and lesions. Histopathological findings of the liver of diabetic rats treated with high dose of betanin were almost similar to that of diabetic control rats (Fig. 2).

#### Pancreas

The pancreatic tissues of normal control and those treated with high dose of betanin showed a normal structure with arranged islets of Langerhans, whereas the pancreas of diabetic control rats exhibited signs of degeneration and inflammation in addition to the decrease in the number of β-cells. Treatment of diabetic rats with 10 and 20 mg/kg of betanin showed efficient regeneration and improvement in pancreatic tissue compared to the diabetic rats treated with high dose of betanin (Fig. 2).

### Gene expression findings

**Fig. 3:**
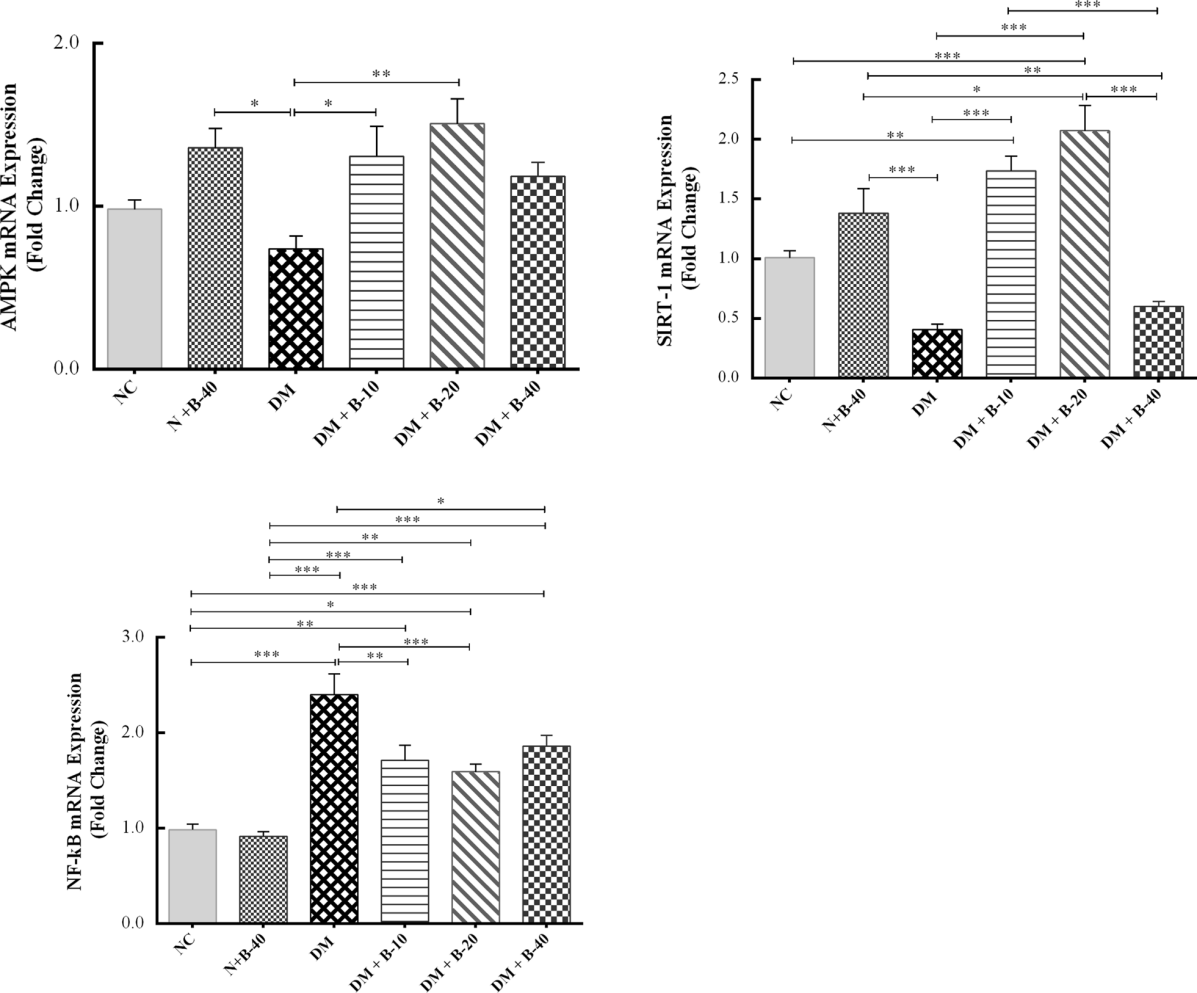
The effects of different doses of betanin on the gene expression of *AMPK*, *SIRT1* and *NF-κB* in the blood of study groups (data are presented as mean ± SEM)

**Fig. 4:**
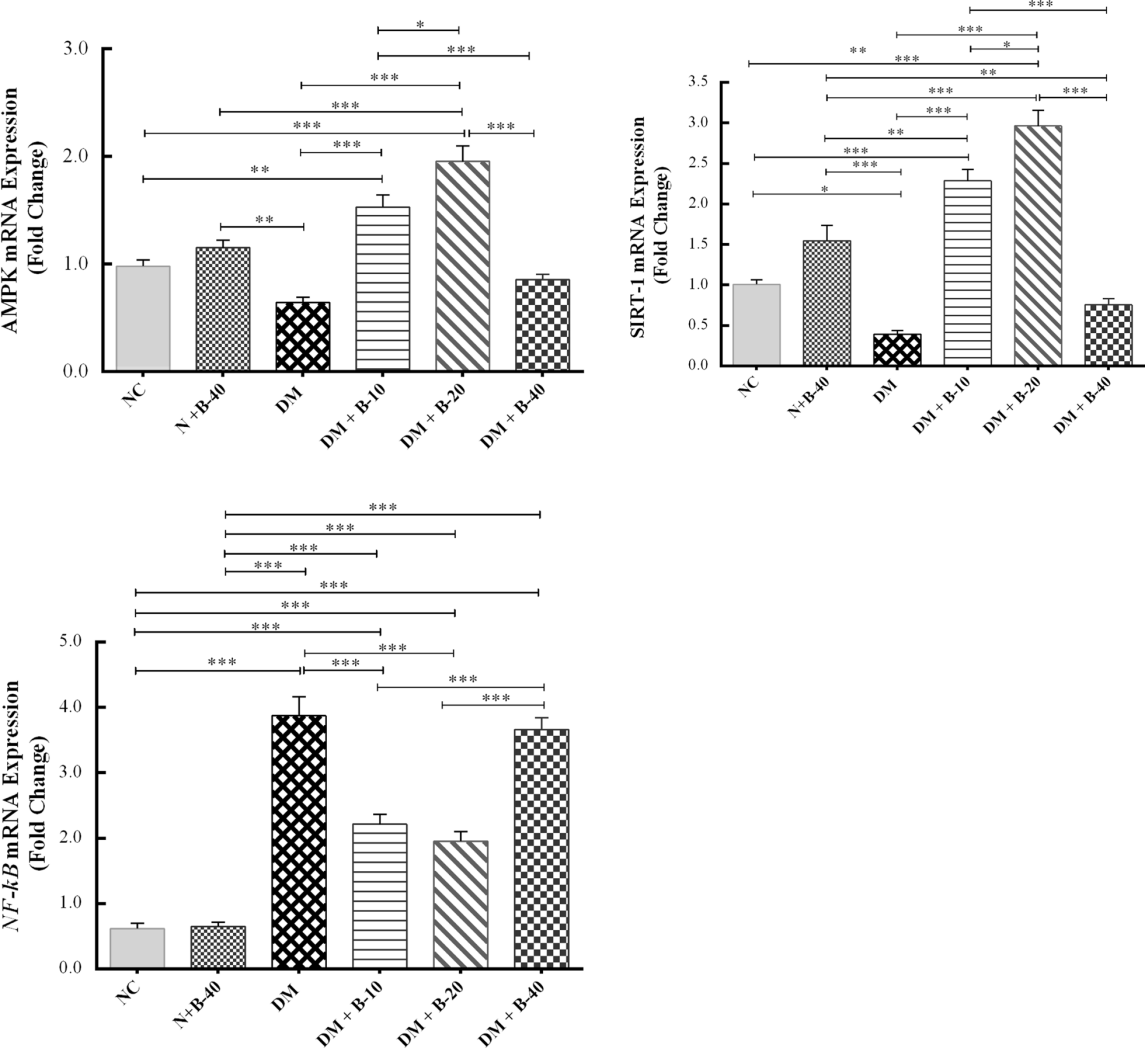
The effects of different doses of betanin on the gene expression of *AMPK*, *SIRT1* and *NF-κB* in the liver of study groups (data are presented as mean ± SEM)

The effects of different doses of betanin treatment on the expression levels of AMPK, SIRT1, and NF-κB were evaluated in whole blood and liver by Real-time PCR. Results of this investigation revealed that treatment of normal rats with high dose of betanin (40 mg/kg) did not have significant effect on gene expression of all the aftermentioned genes in the blood and liver compared to the normal control rats (P > 0.05) (Figs. 3 and 4).

### AMPK

In comparison to the normal control rats, gene expression levels of AMPK in diabetic control rats was reduced in both blood and liver cells, but the difference was not significant (P = 0.756 and P = 0.142, respectively). The treated diabetic rats with low dose of betanin (10 mg/kg) exhibited increased expression of AMPK compared to the diabetic control rats in blood (P = 0.041) and liver tissue (P < 0.001). A similar upward trend was observed in diabetic rats treated with 20 mg/kg of betanin compared to diabetic control rats in the blood (P = 0.003) and in the liver tissue (P < 0.001). Interestingly, the effect of betanin at the dose of 20 mg/kg was significantly more than that of 10 mg/kg of betanin in the liver tissue (P = 0.025).

### SIRT1

The gene expression of SIRT1 in diabetic control rats was reduced compared to the normal control rats. Treatment of diabetic rats with 10 and 20 mg/kg of betanin significantly upregulated the mRNA expression of SIRT1 in comparison to the diabetic control rats (P < 0.001). The effect of the 20 mg/kg of betanin on SIRT1 gene expression seemed to be greater in aall the study groups, especially in the liver (P = 0.014). Betanin at the highest dose did not show any changes in SIRT1expression neither in blood nor in the liver compared to the diabetic control rats (P = 0.927 and P = 0.464, respectively).

### NF-κB

NF-κB mRNA expression was significantly upregulated in the diabetic rats compared to the normal control rats in the blood and liver (P < 0.001). Betanin at different doses could significantly attenuate the expression of NF-κB in the blood (P < 0.05), while in the liver, treatment with the doses of 10 and 20 mg/kg of betanin significantly reduced the gene expression of NF-κB (P < 0.001) compared to the diabetic control rats.

## Discussion

Earlier preclinical studies largely present a promising perspective regarding the potential effect of bioactive food components in preventing and treating metabolic disturbances via different molecular pathways [7]. Betanin, the main natural pigment of red beetroot has been shown to exhibit anti-diabetes properties [39]. In the current study, the effect of different doses of betanin treatment on glycemic control, lipid profile, hepatic function tests as well as histopathological findings of the liver and pancreas tissues were evaluated in STZ-induced diabetic rats. Additionally, the effect of betanin on AMPK, SIRT1, and NF-κB gene expression in the whole blood and liver of the diabetic rats was investigated compared to that of glibenclamide.

Results of the present work showed that STZ injection significantly increased blood glucose levels associated with a significant decrease in insulin levels in diabetic group compared to the control group. Treatment with betanin (10 and 20 mg/kg) and glibenclamide showed decreased FBG levels as well as postprandial glycaemia during a GTT and increased serum insulin levels compared to that of diabetic control. Interestingly, treatment with 20 mg/kg betanin exhibited greater impacts on these factors in comparison with the groups which treated with 10 mg/kg betanin and glibenclamide. GTT has more sensitivity than FBG in the timely assessment of abnormalities in glucose regulation. From the data obtained from GTT, the level of blood glucose reached a peak and returned to fasting values after 2 h in both normal and betanin treated rats (10 and 20 mg/kg) whereas, in diabetic rats, blood glucose levels remained elevated even after 2 h. Betanin effectively prevented the increase of glucose probably due to the enhanced insulin secretion from existing beta cells. STZ induces diabetes by damaging the pancreatic beta cells, reducing insulin secretion and uptake of glucose by the tissues [40]. Based on the previous literature, betanin regenerates the remaining pancreatic beta cells and increases the amount of insulin immunoreactive beta cells, thereby stimulates insulin secretion and improves the level of blood glucose in experimental animals [34, 41]. These finding were supported by the histopathological assay results of our study which showed efficient regeneration and improvement in pancreatic tissue and enhanced insulin secretion after treatment of diabetic rats with 10 and 20 mg/kg of betanin. Sutariya et al. [42] also demonstrated the blood glucose lowering effect of betanin in STZ induced diabetic nephropathy. Similarly, Dhananjayan et al. [34] concluded that the ethanolic extract of beetroot has a potential anti-diabetic activity against STZ induced diabetic rats. According to Olumese and Oboh [43], beetroot juice administration for six weeks led to decreased blood glucose levels by inhibition of carbohydrate digestion and glucose absorption in the intestine as well as the modulation of glucose release from the liver. Additionally, the hypoglycemic activity of the betalains is thought to be attributed to their ability to prevent glycogenolysis and gluconeogenesis [8].

Betanin administration in the present study also led to a significant reduction in TC, TG, and LDL-C levels and a significant increase in HDL-C levels compared to the diabetic control rats. To our knowledge, no previous research is available regarding possible effects of betanin on serum lipid profile in experimental or clinical studies. In an experimental study, the effects of red beetroot crisps (0, 0.3, 1 or 3% of total diet) were investigated in rats with a normal and high-fat diet, and serum TC and TG levels have been reduced at the end of study [44]. In addition, in hypercholesterolemic rats fed with red beetroot extract (250–500 mg/kg), reduced lipid accumulation and significant increase in HDL-C level and antioxidant activity was observed [45].

Some clinical trials also demonstrated the antihyperlipidemic effects of the red beetroot. Holy et al. [46] indicated that red beetroot juice (250 ml) reduced post prandial lipid levels in apparently healthy subjects. Supplementation with betalain-rich extract significantly decreased the concentration of TC, TG, and LDL-C in coronary artery disease patients [47].

Hyperglycemia can induce hepatocellular damages, resulting in elevated liver enzymes including ALT and AST in the blood stream [48, 49]. Results of the present study showed that betanin administration led to a significant reduction in ALT and AST levels, suggesting the tissue protective property of the betanin. As indicated in our histopathological findings, treating diabetic rats with 10 and 20 mg/kg of betanin attenuated the hepatic inflammation and lesions. These results are compatible with Dhananjayan et al. [34] who noticed that the oral administration of betanin could be able to reduce the AST and ALT activities. Decreased liver enzymes levels via the consumption of beetroot have been demonstrated in several other studies [50, 51]. Further evidences support the hepatoprotective effects of beetroot due to the presence of bioactive compounds including betalains as the potent antioxidants [52–55]. Oxidative stress is an important factor in the mechanism of hepatotoxicity and pathogenesis. So, the inhibition of free radicals and/or antioxidant activity plays a crucial role in protecting hepatic injury.

Our findings reveal the antidiabetic, antihyperlipidemic, and hepatoprotective properties of betanin. In order to determine the molecular pathways of these favorable effects of betanin, we further evaluated the effects of different doses of betanin treatment on the gene expressions of AMPK, SIRT1, and NF-κB in the whole blood and liver of the diabetic rats by Real-time PCR.

The study results demonstrated that treatment of diabetic rats with betanin (10 and 20 mg/kg.b.w/day) significantly upregulated the mRNA expression of AMPK and SIRT1 and downregulated the expression of NF-κB in comparison with diabetic control rats.

AMPK serves as a major cellular energy sensor and a master regulator of metabolic homeostasis, where dysregulations in its activity has been reported to contribute to the pathogenesis and progression of metabolic disorders [13]. Some therapeutic alternatives regulate metabolic homeostasis through modulation of different mediators and enzymes, such as AMPK as a potential therapeutic target [26]. AMPK has been demonstrated to upregulate the expression of SIRT1, which regulates various metabolic pathways [15, 56]. Upregulation of SIRT1 has been associated with improved glucose homeostasis, insulin sensitivity, and lipid profile in animal models [57]. SIRT1 is activated downstream, possibly because AMPK activation increases the NAD^+^/NADH ratio, which consequently enhance SIRT1 expression and activation [58]. Besides, there are emerging evidence indicating that the activation of AMPK signaling can inhibit the inflammatory responses induced by the NF-κB system via its downstream mediators like SIRT1, thereby minimizing the inflammatory response [24, 59]. Accordingly, the expression of SIRT1 in peripheral blood mononuclear cells (PBMCs) of patients with coronary artery disease was significantly correlated with inflammatory cytokines levels [28]. Therefore, AMPK and SIRT1 represent as potential targets for the development of therapies to prevent and reduce the incidence of metabolic disease complications and development [26, 57].

Several dietary polyphenols such as flavonoids and resveratrol, alkaloids such as berberine, and some other plant components are potential natural activators of AMPK and SIRT1 [25–27]. It seems that their anti-inflammatory effects are also mediated via the AMPK/SIRT1-induced NF-κB signaling suppress [60].

It has been reported that quercetin, a plant flavonol, increases glucose uptake through stimulation of GLUT4 translocation by activating AMPK [61]. Moreover, a recent study showed that quercetin treatment improved glucose and lipid metabolism and also alleviated hepatic histomorphological injury in STZ-induced diabetic rats, which probably associated with the upregulation of SIRT1 activity by quercetin [62]. Resveratrol, a natural polyphenol, has also been shown to normalize hyperglycemia and hyperlipidemia, improve beta-cell function and insulin sensitivity, and lower hepatic glucose production through the activation of SIRT1 [63, 64]. In another study, the stimulatory effect of hesperetin, a citrus polyphenolic flavonoid, on SIRT1 and AMPK signaling pathway was indicated in HepG2 cells which was even stronger than resveratrol [65]. These findings are consistent with the present study, which reported that the beneficial effects of betanin on glucose and lipid metabolism were enhanced the following increased gene expression of AMPK and SIRT1 in the liver and whole blood of diabetic rats. This finding indicated that modulation of the AMPK/SIRT1/NF-κB signaling pathway by betanin was most likely responsible for its anti-diabetic, anti-hyperlipidemic, and hepatoprotective properties. The relation between AMPK/SIRT1/NF-κB signaling axis and hepatoprotection has been proven in several publication and well-studied. Yu et al. demonstrated that AMPK plays a key role in the hepatoprotective properties of cyanidin-3-glucoside, a natural polyphenol [66]. Similarly, activation of AMPK via a natural polyphenol-rich diet can alleviate alcohol-induced fatty liver in animal model [67]. Moreover, in another animal study, the role of AMPK/SIRT1 signaling axis in hepatoprotection have been confirmed. Ethanol feeding causes alcoholic liver disease (ALD) via the suppression of AMPK activation and cytoplasmic Sirt-1, and administration of dihydromyricetin (5 mg/kg) for 8 weeks can reversed this process [68]. In another study modification of SIRT1by polyphenols was suggested as an important step in the treatment of hepatotoxicity [69].

Despite the numerous studies evaluating the effects of bioactive phytochemicals on cellular metabolic pathways, however, limited evidence is available regarding possible effects of betalains on AMPK and/or SIRT1 levels in diabetes. Kelly et al. [29] reported that the beetroot enriched betalain supplements had significant dose-dependent effects on SIRT1 expression more potent than resveratrol, a well-known SIRT1 activator. Similar results also were found in Rahimi et al. [28] study which showed that treatment with a betalains-rich supplement and particularly a betacyanins-rich supplement significantly increased SIRT1 expression level in PBMCs of coronary artery disease patients. Albalawi et al. [70] showed that administration of beetroot extract led to a significant decrease in NF-kB and TNF-α levels in liver tissue. El Gamal et al. [71] also reported a significant reduction of several pro-inflammatory mediators with oral administration of beetroot ethanol extract in rodents for four weeks.

Our study was initially assumed to consider both low and high doses of betanin on gene expression of AMPK, SIRT1, and NF-κB in STZ-induced diabetic rats. Although betanin augmented SIRT1 expression at the doses of 10 and 20 mg/kg, it was not induced at the higher dose of of betanin (40 mg/kg). This finding suggests that betanin may exert different responses in various doses. Morin et al. [72] defined the biphasic dose responses for nobiletin, a citrus flavonoid, which activated sterol regulatory element-binding proteins at low doses and inhibited it at higher doses. Therefore, it seems likely that betanin has more favorable functions at the lower concentrations.

The current study for the first time indicated that betanin can play its protective roles via the regulation of AMPK/SIRT1/NF-κB signaling pathway in experimentally induced diabetes mellitus. However, as a limitation, the protein levels of AMPK, SIRT1, and NF-κB as well as their activations were not evaluated in this study. In addition, one of the most important end effectors of AMPK/SIRT1 activation in reducing oxidative stress, tissue inflammation, and apoptosis take place by promoting mitochondrial biogenesis and alteration in the mitochondrial physiology [73]. This aspect was also not addressed in the present study and requires further investigation. It is also suggested that in the future researches, the effect of betanin on eating behaviors such as food and water intake, frequency of food intake, adipokine levels such as ghrelin, leptin and adiponectin in STZ-induced diabetic rats should be investigated.

## Conclusion

In conclusion, the results of the present study demonstrated that betanin exertes beneficial effects against diabetes complications in the STZ-induced diabetic rats. These effects are mediated by reducing hyperglycemia, hyperlipidemia, and liver functional tests as well as improving liver and pancreas tissues functionality. These promising effects of betanin were demonstrated to be mediated through the AMPK/SIRT1/NF-κB signaling pathway. Our findings support the theory by which betanin have been recognized as an effective nature-inspired bioactive ingredient in the management of metabolic disorders such as diabetes mellitus. However, future studies are needed in order to further establish the potential protective effects of betanin to overcome metabolic diseases.

## Data Availability

All data of present study are available from the corresponding author on reasonable request.

## Abbreviations

ALT: Alanine aminotransferase
AMPK: 5′ Adenosine monophosphate‑activated protein kinase
ANOVA: Analysis of variance
AST: Aspartate transaminase
CAD: Coronary artery disease
DM: Diabetes mellitus
ELISA: Enzyme-linked immunosorbent assay
FBG: Fasting blood glucose
GTT: Glucose tolerance test
HDL-c: High-density lipoprotein cholesterol
NADH: Nicotinamide adenine dinucleotide
NF-κB: Nuclear factor kappa B
PBMC: Peripheral blood mononuclear cell
PCR: Polymerase chain reaction
SIRT1: Sirtuin 1
STZ: Streptozotocin
TC: Total cholesterol
TG: Triglyceride
